# Optimization of English Machine Translation by Deep Neural Network under Artificial Intelligence

**DOI:** 10.1155/2022/2003411

**Published:** 2022-04-21

**Authors:** Xiaohua Guo

**Affiliations:** College of Translation Studies, Xi'an Fanyi University, Xi'an 710105, China

## Abstract

To improve the function of machine translation to adapt to global language translation, the work takes deep neural network (DNN) as the basic theory, carries out transfer learning and neural network translation modeling, and optimizes the word alignment function in machine translation performance. First, the work implements a deep learning translation network model for English translation. On this basis, the neural machine translation model is designed under transfer learning. The random shielding method is introduced to implement the language training model, and the machine translation is slightly adjusted as the goal of transfer learning, thereby improving the semantic understanding ability in translation performance. Meanwhile, the work design introduces the method of word alignment optimization and optimizes the performance of word alignment in the transformer system by using word corpus. The experimental results show that the proposed method reduces the average alignment error rate by 8.1%, 24.4%, and 22.1% in EnRo (English-Roman), EnGe (English-German), and EnFr (English-French), respectively, compared with the previous algorithms. Compared with the designed optimization method, the word alignment error rate is lower than that of traditional methods. The modeling and optimization method is feasible, which can effectively solve the problems of insufficient information utilization, large parameter scale, and difficult storage in the process of machine translation. Additionally, it provides a feasible idea and direction for the optimization and improvement in neural machine translation (NMT) system.

## 1. Introduction

As a bridge of communication, in the process of globalization in modern society, language has become a necessary condition for human beings to exchange information. As an important means of cross-language communication, translation plays a very vital role [1]. In the era of rapid interaction of language and information, human translation can no longer meet the high-speed cross-language communication. The focus of translation has gradually evolved to be based on high-tech artificial intelligence (AI) technology. With the development of Internet and computer, MT is gradually improved and widely used [2]. Machine translation (MT) methods are generally divided into three types: rules, examples, and statistics. Before 1980s, rule method dominated the field of MT. This method generally uses vocabulary rules, conversion rules, and syntax generation rules and derives techniques such as text processing, syntax analysis, and dictionary [3, 4]. However, as the amount of information in the language system increases and the speed of information interaction increases, the limited rules and complicated operations established manually in the rule method gradually fail to adapt to the new environment of language information [5]. The MT technology published in 1980s is different from the rule technology in that it relies more on bilingual corpus inventory, and the target language is output through matching in corpus. The latter method also has some limitations, such as high labor cost and low matching rate. The essence of statistical translation method is to collect and statistically analyze bilingual corpus. Due to the sparsity of data, this method has obvious defects when dealing with sentences that depend on long distance and paragraphs that have strong contextual relevance.

In 1989, Zhang et al. proposed convolutional neural network (CNN). With the enhancement of computer computing ability, the performance of CNN is also improving. At present, CNN is considered to be one of the most widely used machine learning technologies, especially in computer vision tasks. CNN's strong learning ability is mainly due to the use of multiple feature extraction layers, which can automatically learn discriminative high-level semantic features from data [6]. Typical CNN architecture usually includes alternately stacked convolution layer and pooling layer, and the top is the classification or regression layer composed of one or more fully connected layers. In addition to using different activation functions, batch normalization and dropout regularization are also used to optimize the performance of CNN [7]. At present, MT has been widely used in the fields of medical treatment, law, etc. In order to cope with the different language environments of different users, MT not only meets the translation needs of products, but also has the characteristics of short translation cycle [8]. Neural machine translation (NMT) gradually becomes an important research field in MT [9], thanks to its advanced translation performance. The neurons of neural network are similar to those of the human brain. When neurons receive signals from different body parts, they will reflect different signals [10], and the neural network operates in a similar way, where it receives the input of information and generates output and realizes the final output after transmitting it layer by layer. At present, NMT has achieved remarkable results in a short time. In the performance research and experiment of NMT by global researchers, it has been proved that NMT technology is equal to or superior to statistical MT in more than 30 translation directions, and NMT can jointly train multiple features without prior knowledge. After optimizing the sentences, the NMT can get translation results [11, 12]. NMT can solve the problems of word order errors and syntactic and morphological errors in translation, and its memory requirement is smaller than traditional methods. However, NMT has many parameters and is difficult to optimize. Meanwhile, it is difficult to produce high-quality alignments. Therefore, the transformer system is taken as the benchmark. Through the research on optimization of multilayer network, storage difficulty of parameter scale, and alignment method, the research serves to optimize NMT system and provides ideas for solving above problems.

Although in terms of effect, NMT has indeed made great progress, there are still some problems: (1) Incomplete dictionary NMT is based on neural network, and its dimension is directly affected by the dictionary size. In order to save space and shorten time, the dictionary size is generally less than 50000 and common about 30000. Unlisted words will be replaced with UNK (Unknown) identifier during translation. However, in this way, the source language information will be difficult to be accurately and completely captured. As for the target language side, the existing UNK identifier will also affect the overall effect of the sentence, making it more difficult for people to accurately understand the output sentence. The richer the part of speech, the more prominent this problem is. (2). The attention mechanism of over translation and omission is a particularly important point in NMT. In the decoding process, the target words are flexibly output by calculating the attention of the target language to each word on the source language at each step. However, there is no auxiliary information in each attention calculation, and there may be a problem that a source language word is highly paid attention to many times or the attention of some words is too small. (3). The semantics of the translation is inaccurate. The word representation in NMT adopts the method of continuous representation. While improving the generalization effect of translation model and the fluency of translated sentences, it is also easy to cause the problem of inaccurate semantics of the translation. In order to better solve the above problems, the work studies and designs the deep neural network (DNN)-based machine translation technology model, adds a migration learning algorithm, and uses the random shielding method to establish the pretraining model based on encoder-decoder to realize the model prediction of the network. Meantime, a word alignment optimization method is designed on the transformer system. The innovation is to propose a NMT method based on transfer learning. In this method, the end-to-end encoder-decoder framework language pretraining model is constructed by using random shielding, which shields the random continuous character segments in the input of the source language sequence, and then predicted by the network model. Then, on this basis, machine translation is fine-tuned as the target task of transfer learning, and the prior knowledge in the pretraining language model is applied to the target task. The contribution is to design a vertical domain-oriented NMT method based on transfer learning, which improves the semantic understanding and comprehensive performance of NMT.

## 2. Design of MT Modeling and Optimization Method Based on DNN

### 2.1. Neural Network Translation Modeling

#### 2.1.1. Encoder and Decoder

The encoder is usually composed of a neural network model. The encoder can process the input information, map the variable length source language in the encoder into a fixed length state vector containing context information, and encode the information of the input sequence in the state vector [4, 13].

**Figure 1:**
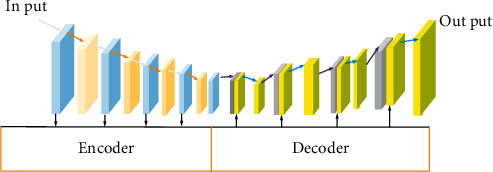
Schematic diagram of encoder and decoder.

The decoder is composed of neural network. The ultimate goal is to obtain the output sequence with the highest probability. The higher the probability of the sequence, the more reasonable the possibility of the sequence [14–16]. In the process of decoding, the decoder will search for the maximum conditional probability. The two common methods are exhaustive search and greedy search [17]. The process of exhaustive search is to enumerate all probabilities, and the optimal sequence is obtained by maximum probability. The disadvantage of this method is that when the thesaurus is too large, the computational overhead will also increase. The process of greedy search is to find the maximum probability of the current time sequence and obtain the local optimal solution [18]. The above equations suggest that the propagation process of recurrent neural network (RNN) includes the steps of chain derivation. Due to the existence of network depth and continuous multiplication formula, gradient explosion or disappearance may occur in the process. This problem can be effectively solved by gradient clipping, that is, setting the threshold of gradient vector. The schematic diagram of encoder and decoder is shown in Figure 1 below:

#### 2.1.2. Attention Mechanism in MT

**Figure 2:**
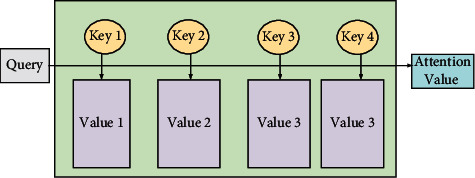
Attention mechanism structure and process.

The attention mechanism, first used in image classification task, has been embedded into a variety of neural networks [19]. In the process of natural language generation (NLG) machine translation or text summarization, the occurrence frequency of long sequence text is relatively high, and the relationship between context is relatively close [20]. When using fixed length coding to represent more complex and long sequences, it is easy to lose the input information. For example, the working structure of two RNNs is difficult to reflect the relationship between sequences [21]. Introducing the attention mechanism can allow the decoder to access the coding vector generated by the encoder, reorganize the weight, and finally obtain the output results. This process can effectively solve the loopholes and defects of long coding input and output [22]. The attention mechanism is mainly a mechanism that focuses on specific parts of the source language sequence in each time sequence of the encoder [23]. The MT model introducing the attention mechanism can achieve better relationship attention to indefinite length sequences. Each time sequence in the decoder gives different weight values of attention to the information encoded at different times, resulting in multiple context vectors. The output sequence is obtained according to the weight value, to improve the restoration degree of content and the fluency of sentence grammar. The process modeling of the attention mechanism is shown in Figure 2.

As Figure 2 shows, the essential idea of the attention mechanism is to treat the constituent elements in the input as key values to form the data, and the key values to the data correspond to the query element in the output. By calculating the similarity between the query element and each key, the weight coefficient of the key value corresponding to each key can be obtained, and the value of the final attention mechanism can be obtained by summing multiple key values. The original encoder-decoder coding model applied in RNN does not consider the attention weight. After the introduction of attention, the encoder-decoder coding model will determine which input should receive more attention according to the previous output before each output.

#### 2.1.3. Structure of the Transformer MT System

The transformer system is the mainstream framework of neural network MT. As one of the advanced NMT systems, the transformer system models through the sequence of self-attention mechanism [24], and models through the source-end self-attention mechanism and the target-end self-attention mechanism, and obtains the source language sequence represented by equation (1) and the target language sequence represented by equation (2), thus obtaining the representation of corresponding information.

(1) $$\begin{array}{c} X=\left(x_{1},x_{2},\ldots ,x_{m}\right), \end{array}$$

(2) $$\begin{array}{c} Y=\left(y_{1},y_{2},\ldots ,y_{m}\right). \end{array}$$

The encoder of the transformer system is composed of *N* layers of networks stacked, and each layer in *N* layers of networks is composed of a self-attention mechanism sublayer and a feedforward neural network sublayer. Compared with the encoder, the decoder stacked by *N* layers of networks also has an attention sublayer. To solve the problem that the local numerical gradient is too small, the transformer system adopts the attention mechanism of scaling points. In the encoding stack, the last encoder passes the vector of the output state to all decoders in the stack end of decoders, which is used as the encoding input corresponding to the context. The vector output by the decoder and the output information at the upstream of the encoder are taken as their own input information and are used for probabilistic output [25, 26].

### 2.2. Optimization Method Design of Neural Network Word Alignment

#### 2.2.1. Alignment Network Modeling

**Figure 3:**
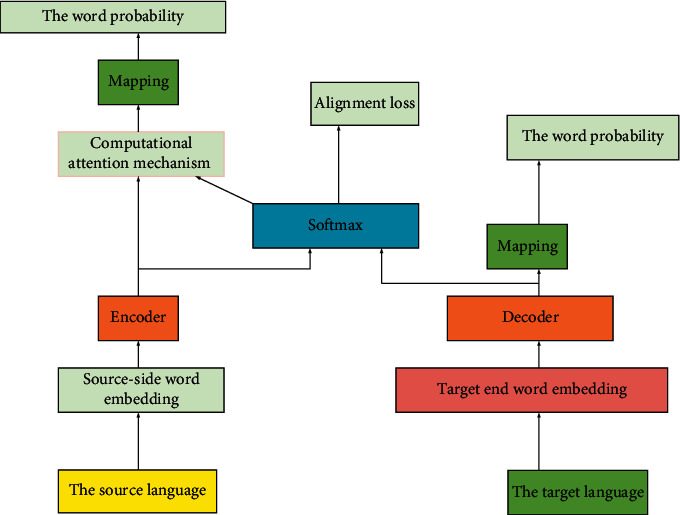
Alignment network optimization model.

There are generally two methods for word alignment optimization. First, initializing the linear mapping matrix before the output of the transformer system and alignment network by using single language data pretraining, thus enhancing the expression of source language vocabulary and target language vocabulary in the model [27]. Second, reducing the confusion of the corresponding relationship between the source language vocabulary and the target language vocabulary by aligning the confusion of the regular items in the set. The alignment network optimization model is presented as Figure 3.

#### 2.2.2. Design of Optimization Method

The word alignment in the traditional method requires the whole sentence to be generated before the word alignment of the downstream task. In the MT process, every target word generated by the translation behavior should be aligned to the position of the maximum attention weight in the alignment layer. In the process of translating words one by one, attention weight will be affected by context information in different time series. A cross-linguistic language model XLM based on single corpus of different languages is introduced [28]. Concurrently, byte pair encoding (BPE) based on joint language and target language can obtain a shared vocabulary used in XLM model, translation model, and alignment network [29]. Parameter *C* is defined as the association between the source language data with the target language data, *i* stands for the character position that can be selected, *M* is the masked set of positions, and *θ* refers to the model index, and there are three situations that can replace the *I* character, which are as follows: *A*: a probability of 80% of replacing “test”; *B*: a probability of 10% of replacing random characters; and *C*: 10% probability of not replacing. By using “test” as a word for training, the equation of loss function can be obtained as shown in (3).

(3) $$\begin{array}{c} L\left(\theta ;C\right)=\frac{1}{C}\sum_{c\in C}\sum_{m\in M}\log\;P\left(c_{m}|\overset{⌢}{c};\theta \right). \end{array}$$

For the attention mechanism of the target language, the higher the weight value of the source position, the greater the impact on the target vocabulary. Usually, the vector obtained after modeling the internal dependency of sentences in the translation process will lead to the weight value in the attention mechanism being scattered to multiple positions [30]. Because word alignment represents the corresponding relationship between the source language vocabulary and the target language vocabulary, the weight distribution will affect the realization quality of word alignment. A method is designed to measure the degree of attention confusion by using entropy and improve the quality of word alignment by controlling and optimizing the distribution of attention weights. Entropy is a standard to measure the degree of attention confusion. The higher the entropy, the higher the degree of confusion, and the lower the reliability. Parameter *β*_*t*,*a*_ is defined as the attention weight when *t* is at the *i* position of the source end of the alignment layer, and the loss function is obtained as follows:

(4) $$\begin{array}{c} L_{\text{con}}^{t}=-\sum_{a=1}^{m}\left(\beta_{t,a}\log\right.\left(\beta_{t,a}\right). \end{array}$$

In the above equation, the confusion of word alignment is reduced by minimizing the loss of concentration. For the newly added loss of regular items in its set and the loss of vocabulary prediction in the alignment layer, a balance factor *λ* is introduced to adjust. Defining *L*_con_^*t*^ as the loss of central regularization and *L*_word_^*t*^ as loss of vocabulary prediction, the importance of the two parameter is expressed as follows:

(5) $$\begin{array}{c} L=L_{\text{word}}^{t}+\lambda L_{\text{con}}^{t}. \end{array}$$

**Figure 4:**
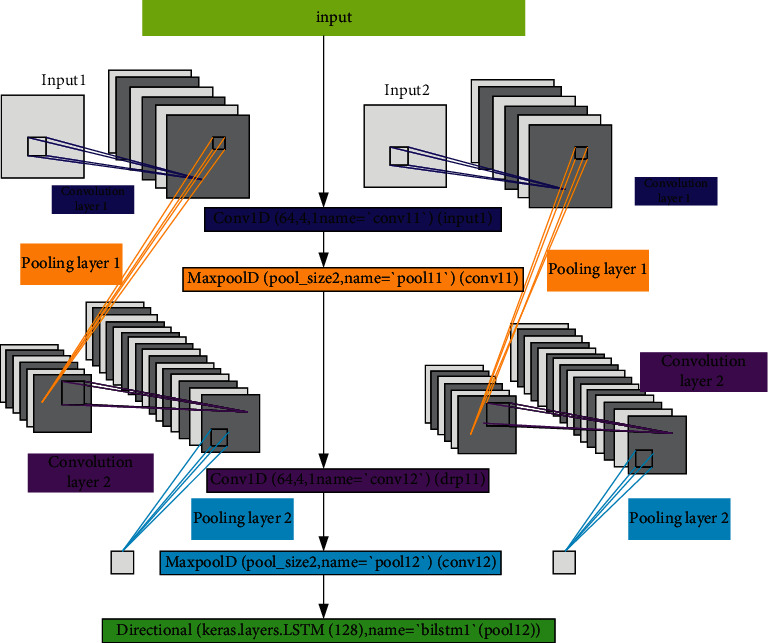
The implemented model.

Figure 4 is the model implemented here.

### 2.3. Experiment Design and Data Integration

#### 2.3.1. Data Sources

For the experiment of neural network MT model, the research selects data from the 2020 International MT Competition and Internet-related public data and sets up a training data group with 500,000 lines, a control data group with 2,000 lines, and two test data groups with 1,000 lines.

For the experiment of transfer learning, the selected data come from the 2020 International MT Competition and Internet-related public data as a set of training data with a total of 1,000,000 lines, a set of class A fine-tuning data with a total of 250,000 lines, a set of control data with a total of 1,000 lines, a set of Class B fine-tuning data with a total of 250,000 lines, and a set of control data with a total of 1,000 lines.

#### 2.3.2. Development Indexes

Python3.7 is selected as the development language. TensorFlow and Tensor2Tensor are applied as the deep learning framework.  Table 1 lists the index settings.

Table 1 suggests that the size of synonym dictionary used in this experiment is 8000, the dimension of word vector is 512, the batch size is 1024, the number of network layers is 6, the Dropout rate is 0.2, and the learning rate is 0.1. The optimizer used here is Adam optimizer.

Table 2 presents the experimental index settings for transfer learning.

As Table 2 presents, in the transfer learning, the work selects the synonym dictionary size of 16000, the word vector dimension of 512, the batch size of 1024, the dropout rate of 0.1, the learning rate of 0.1, and the optimizer is Adam optimizer.

Table 3 displays the experimental index settings for word alignment optimization.

Table 3 indicates that, when optimizing learning, the work selects synonym dictionary size of 8000, word vector dimension of 512, batch size of 1024, dropout rate of 0.1, learning rate of 0.0005, and Adam optimizer.

## 3. MT and Test Results for Optimization

### 3.1. Examination of Results of Neural Network MT

**Figure 5:**
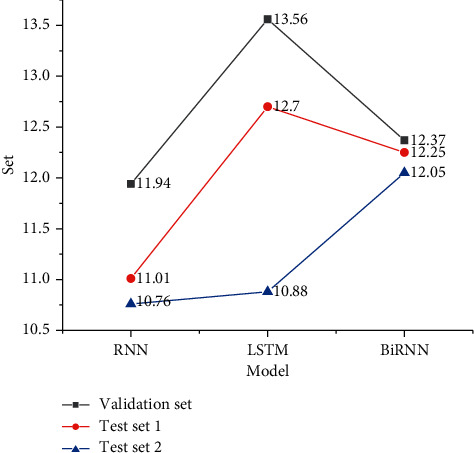
Score results without the attention mechanism.

**Figure 6:**
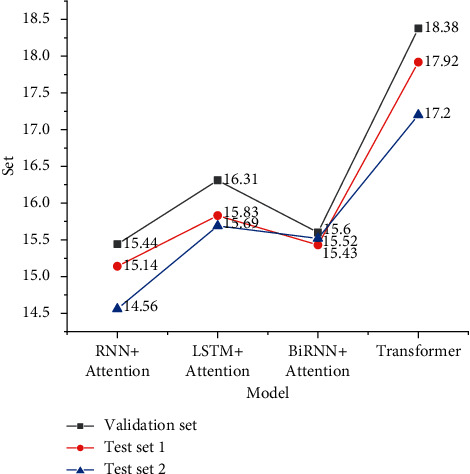
Score results with the attention mechanism.

Based on the above development indexes and data settings, three models, RNN, long and short-term memory (LSTM), and bidirectional recurrent neural network (BiRNN), are trained and tested again after adding the attention mechanism. Finally, the transformer model is compared, and Figures 5 and 6 signify the experimental results.

Figures 5 and 6 show that the MT model designed here is feasible, the scores exceed the baseline level, and the score of the transformer system is the highest. In the model test with the attention mechanism, LSTM has a higher score level and the best translation performance, which is 15.83; BiRNN has a better performance, which is 15.69, and RNN has the weakest performance among the three. After adding the attention mechanism calculation, the translation performance of the three models is improved. Compared with the three models, the transformer's model structure can reduce the computational complexity and realize the computational parallelism of the encoder, so the performance improvement effect is more obvious. The neural network with the attention mechanism can focus the context information on the content strongly related to the current generated sequence through the attention matrix and attention weight. It can also reflect the global to local relationship, improve the disadvantage of information stacking in the forward neural network, and further improve the performance of the network model from the perspective of reduction and fluency.

**Figure 7:**
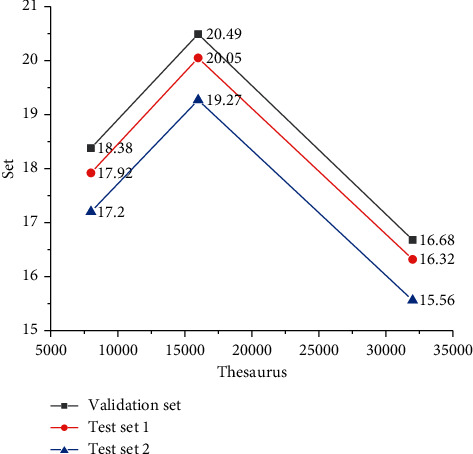
Comparison test of vocabulary.

Based on the above results, taking the transformer as the experimental model, the experiment is carried out to compare the indexes of thesaurus size.  Figure 7 gives the results.

In Figure 7, when the number of tests is 10000, the first test result of the model is 17.92 and the second test result of the model is 17.2; when the number of tests is 20000, the first test result of the model is 20.05 and the second test result of the model is 19.27, and the performance of the model is the best. When the number of tests is 30000, the performance of the model decreases.

It can be explained that the size of thesaurus has a certain impact on the performance of NMT model, but it does not increase linearly with the size of thesaurus. When the size of thesaurus exceeds a certain size, it reduces the model performance. The reason may be the limitations of the treatment of rare words. For example, when the thesaurus setting is too large, some illegal characters will be added to the thesaurus, which will affect the translation performance. Therefore, it can be concluded that appropriately adjusting the size of thesaurus in a certain range according to the size of data set is helpful to improve the performance of the model.

### 3.2. Test Results of Transfer Learning Model

**Figure 8:**
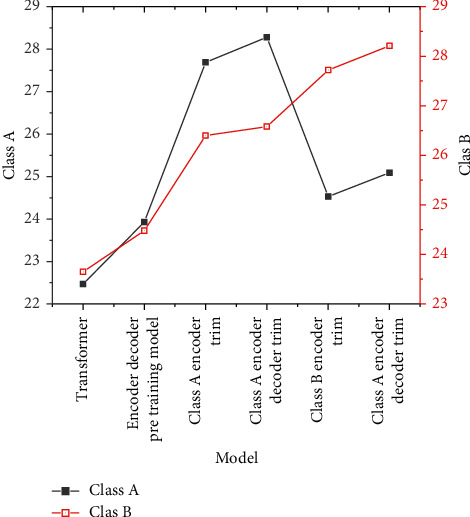
Test results.

By using the pretraining model designed above, the transformer system with high performance is selected as the experimental system, and the pretraining model is tested in the data set after fine-tuning the class A and class B corpora.  Figure 8 displays the experimental results of class A and class B corpora.

The test results show that the pretraining model of encoder-decoder gets the highest score, which can significantly improve the performance of MT. Additionally, the data results after fine-tuning show a certain upward trend in the performance score, so the fine-tuning of the pretraining model is feasible and valuable.

**Figure 9:**
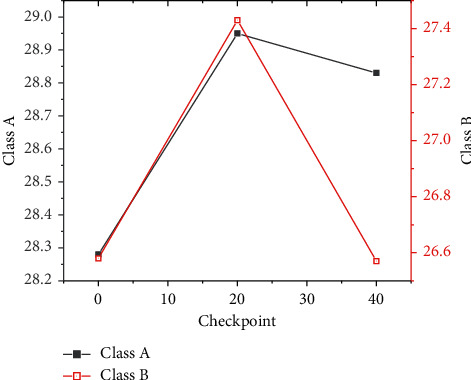
Checkpoint preservation experimental results.

For the research of checkpoints, the model of class A encoder-decoder fine-tuning is chosen for experiments, and checkpoint preservation is performed every 2000 steps in iterative training.  Figure 9 shows the experimental results.

According to the results in Figure 9, the weight of class A data is increased by 0.75 points when it is saved at 20 checkpoints and 0.34 points when it is saved at 40 checkpoints. The weight of class B data is increased by 0.45 points when it is saved at 20 checkpoints and 0.44 points when it is saved at 40 checkpoints. It is not difficult to see that the average of checkpoints can effectively solve the over-fitting phenomenon of data. Therefore, the average operation of checkpoints positively impacts the improvement in translation performance. But when a certain threshold is reached, the impact will become negative, which may be caused by too many nodes and polluted parameters in the iterative process.

### 3.3. Word Alignment Optimization Test

**Figure 10:**
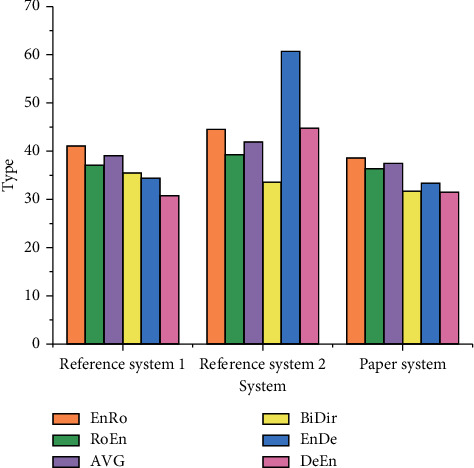
EnRo to GeEn word alignment error rate without dynamic evaluation.

**Figure 11:**
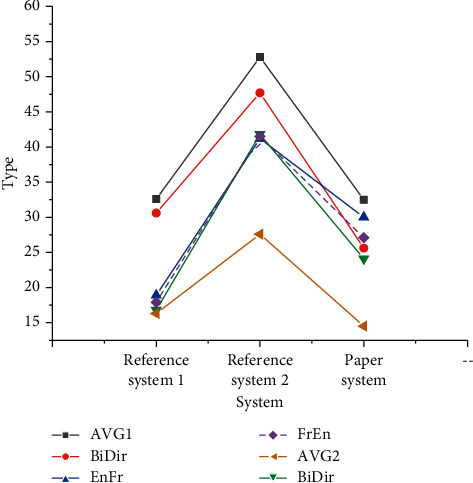
AVG to BiDir word alignment error rate without dynamic evaluation.

For the sentence-by-sentence word alignment of the test set, AVG represents the average error rate of two directions in a single data set, BiDir represents the final alignment of two directions on a single data set by heuristic method and the inapplicability dynamics of EnRo (English-Roman), RoEn (Roman-English), EnGe (English-German), GeEn (German-English), EnFr (English-French), and FrEn (French-English). EnRo to GeEn word alignment error rate without dynamic evaluation and AVG to BiDir word alignment error rate without dynamic evaluation is shown in Figures 10 and 11, respectively

**Figure 12:**
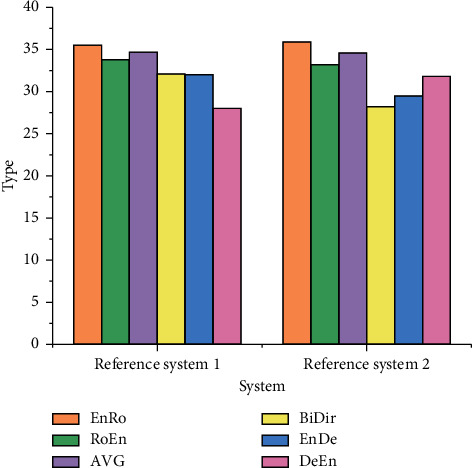
EnRo to GeEn word alignment error rate with dynamic evaluation.

**Figure 13:**
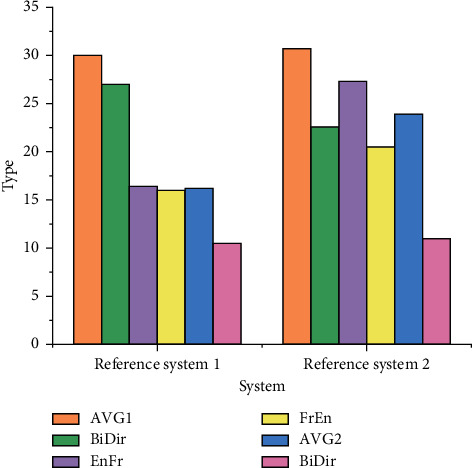
AVG to BiDir word alignment error rate with dynamic evaluation.

Figures 10 and 11 reveal that the error rate of the design scheme in the translation data set is lower than that of Reference system 1 as a whole, and the error rate of EnDe data set is 24% lower as a whole. The error rate test results using the dynamic evaluation method are shown in Figures 12 and 13.

After adding dynamic evaluation, the designed method shows a low error rate on EnDe and other corpora, and the transformer's neural network translation method shows the best combined result. The reasons are as follows: multitasking learning is equivalent to increasing the sample size for training model. Because all samples are not distributed in the ideal state, there is some noise, and there is a risk of over-fitting when training a separate model. Multitask learning under different samples can balance these noises, and sharing parameters weakens the network ability to a certain extent, reduces the possibility of task over-fitting, and improves the generalization ability of the model. In addition, multitask learning can further deepen the model's attention to the common characteristics of multiple related tasks. The way of global weight sharing can make the model pay more attention to hidden layer information, mine hidden patterns at different task levels, and apply them to main tasks.

## 4. Conclusion

Language is the most important bridge in communication. The exchange and translation of global languages has become an indispensable condition for human friendly exchanges all over the world. As the oldest communication activity in the history of human development, translation now occupies a pivotal position in the process of global integration. With the increasing maturity of computer technology and the rapid change in the Internet, MT technology has gradually stepped into the stage of history. MT has moved from the academic discussion to the industrial application, which is of great significance for the research on MT. Based on the deep learning network, the work studies the English-Chinese NMT model. The work mainly discusses and designs the MT technology model based on DNN, adds a transfer learning algorithm, and uses the random shielding method to implement the pretraining model of the encoder-decoder to realize the model prediction of the network. Moreover, a word alignment optimization method is designed on the transformer system. Experiments show that the designed method has better word alignment performance than the traditional methods, which improves the overall performance level of MT. Based on deep learning network, the work studies English-Chinese NMT model and has achieved some results, but there are still many deficiencies. In the later learning process, there is still a lot of work to be completed: (1) due to the limited experimental cost, the model training level is far lower than the cutting-edge standard. Whether due to the scale of training data or some parameters, it cannot be used to maximize to improve the model performance. (2) There are still many details worth exploring in MT research, such as the research of domain adaptation and the improvement in unsupervised training performance.

## Figures and Tables

**Table 1:** Experimental indexes of translation model.

Index type	Indexes
Thesaurus size	8000
Word vector dimension	512
Batch size	1024
Number of network layers	6
Dropout	0.2
Learning rate	0.1
Optimizer	Adam
Beam search width	5

**Table 2:** Experimental indexes of transfer learning.

Index type	Indexes
Thesaurus size	16000
Word vector dimension	512
Batch size	1024
Fine tuning iteration steps	200000
Dropout	0.1
Learning rate	0.1
Optimizer	Adam
Beam search width	5

**Table 3:** Experimental indexes of word alignment optimization.

Index type	Indexes
Thesaurus size	8000
Word vector dimension	512
Batch size	1024
Number of network layers	6
Dropout	0.1
Learning rate	0.0005
Optimizer	Adam
Beam search width	5

## Data Availability

The data used to support the findings of this study are included within the article.
